# The Role of Autophagy in Crohn’s Disease

**DOI:** 10.3390/cells1030492

**Published:** 2012-08-03

**Authors:** Paul Henderson, Craig Stevens

**Affiliations:** 1 Department of Child Life and Health, 20 Sylvan Place, University of Edinburgh, Edinburgh EH9 1UW, UK; 2 Gastrointestinal Unit, Institute for Molecular Medicine, University of Edinburgh, Western General Hospital, Crewe Road, Edinburgh EH4 2XU, UK; Email: C.Stevens@napier.ac.uk; 3 Faculty of Life, Sport and Social Sciences, Edinburgh Napier University, Sighthill Campus, Edinburgh EH11 4BN, UK

**Keywords:** autophagy, Crohn’s disease, inflammatory bowel disease, ATG16L1, NOD2, IRGM

## Abstract

(Macro)-autophagy is a homeostatic process by which eukaryotic cells dispose of protein aggregates and damaged organelles. Autophagy is also used to degrade micro-organisms that invade intracellularly in a process termed xenophagy. Genome-wide association scans have recently identified autophagy genes as conferring susceptibility to Crohn’s disease (CD), one of the chronic inflammatory bowel diseases, with evidence suggesting that CD arises from a defective innate immune response to enteric bacteria. Here we review the emerging role of autophagy in CD, with particular focus on xenophagy and enteric *E. coli* strains with an adherent and invasive phenotype that have been consistently isolated from CD patients with ileal disease.

## 1. Introduction

Crohn’s disease (CD), one of the major forms of inflammatory bowel disease (IBD), is a chronic disease of the intestinal tract associated with significant morbidity [1]. CD may occur anywhere in the gastrointestinal tract with discontinuous transmural inflammation, commonly affecting the terminal ileum (distal small bowel) in adults, but with more extensive and often panenteric involvement in children [2]. The exact etiological factors involved in IBD aetiopathogenesis remain elusive; however it is clear that genetic predisposition [3], environmental influences [4] and a dysregulated immune response to the intestinal microflora are involved [5]. Major CD susceptibility pathways uncovered through recent genome-wide association studies (GWAS; the examination of many common genetic variants in different individuals to determine any association with a trait) implicate the innate immune response (e.g., NOD2), the more specific, acquired T cell response (e.g., *IL23R*, *ICOSLG*) and more recently autophagy (e.g., ATG16L1, IRGM) [3,6]. Examination of the disease-associated microbiome has also alluded to several potentially contributory organisms, most notably adherent-invasive *E. coli* strains (AIEC), which have been isolated by independent investigators in both adult [7] and paediatric [8] CD patients. This review will discuss the emerging role of autophagy in CD, with particular focus on enteric *E. coli* strains with an adherent and invasive phenotype (AIEC).

## 2. (Macro)-Autophagy

Two cellular processes, the ubiquitin-proteasome pathway and autophagy are involved in the removal of redundant, misfolded and harmful proteins [9,10]. Unlike the proteasome, autophagy can target large protein aggregates and also has the ability to degrade deoxyribonucleic acid (DNA), ribonucleic acid (RNA) and lipids [11]. Ultimately all autophagic pathways converge at the lysosome, a central, acidic organelle that harbours lysosomal hydrolases such as peptidases and nucleases [12]. Three major types of autophagic mechanisms have been described in mammalian cells, namely chaperone-mediated autophagy (CMA), microautophagy and macroautophagy. CMA is estimated to degrade approximately 30% of cytosolic proteins, specifically targeting proteins bearing a motif in their amino acid sequence biochemically related to the pentapeptide KEFRQ [13]. This pathway is mediated by heat shock cognate 70 and degradation occurs following interaction with lysosomal-associated membrane protein 2 [14]. Microautophagy refers to the direct engulfment of the cytoplasm by the lysosome [15]. This process has been shown to occur in five main stages, namely microautophagic invagination, followed by vesicle formation, expansion, scission and degradation [15]. Although discovered in the 1960s [16], many of the mechanisms involved in microautophagy are still unclear and its role in disease is still to be determined. Although several other forms of autophagy are now being investigated [15], it is the process of macroautophagy which has gained most attention in the field of disease pathogenesis [17]. Macroautophagy (hereafter referred to as autophagy) differs from CMA and microautophagy in that it relies on specialised double membrane vesicles called autophagosomes that form *de novo* through the assembly of protein and lipid constituents. Enigmatic for many years, it is now clear that autophagosomes originate from the surface of membranes of various cell organelles, including the endoplasmic reticulum, mitochondria and the plasma membrane [18]. Autophagy is upregulated in response to many stimuli including starvation, genotoxic stress and microbial infection [19]. 

**Figure 1:**
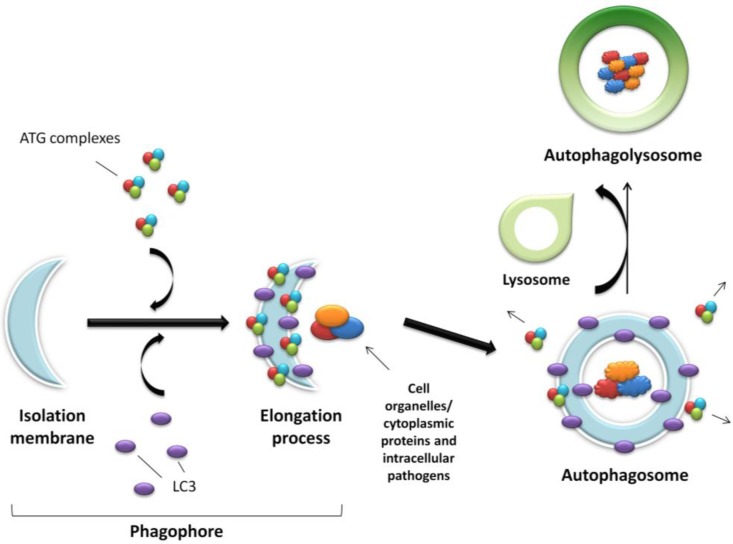
Schematic representation of autophagy. An expanding membrane sac (the phagophore) sequesters cytosolic material forming a double membrane vesicle (the autophagosome) enclosing proteins, organelles or pathogens to be degraded. The autophagosome then fuses with the acidic lysosome forming the final autophagolysosome.

The process of autophagy is controlled by the ATG proteins (or autophagy-related proteins) and can be divided into three distinct stages: vesicle nucleation, vesicle elongation and fusion of the autophagosome with a lysosome (Figure 1).

**Figure 2:**
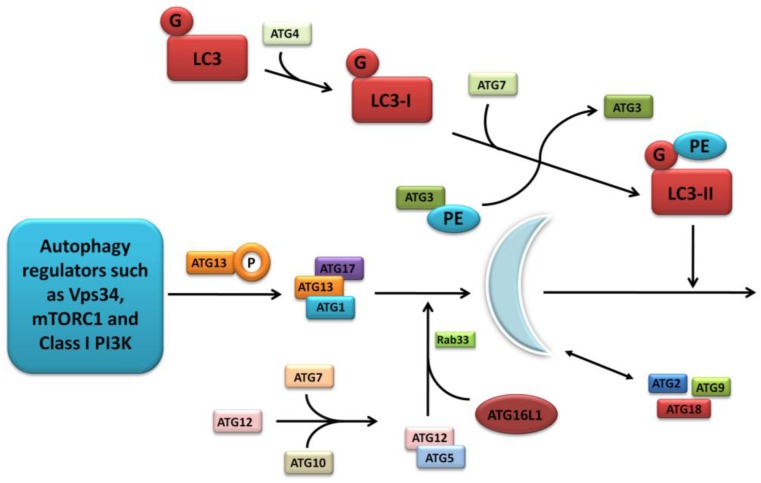
Molecular machinery of autophagosome formation. Two ubiquitin-like protein conjugation systems (the LC3/ATG8 and ATG12 systems) mediate membrane formation and expansion. PE, phosphatidylethanolamine; G, glycine.

The further detailed and complex molecular machinery involved in autophagosome formation is outwith the scope of this focussed review, however a summary of the major pathways are outlined in Figure 2 and discussed comprehensively in several excellent articles [17,20,21].

## 3. Antimicrobial Autophagy (Xenophagy)

Autophagy is activated in response to diverse stress or stimuli, including infection and is a crucial part of the innate immune defence against micro-organisms [22]. Antimicrobial autophagy, also referred to as xenophagy can selectively target microbes, including virus, protozoa and bacteria for degradation in lysosomes [22]. Host cell defence against infection is complex and involves the convergence of several pathways. Pathogens invading host cells are initially detected by pattern recognition receptors (PRR), which include Toll-like receptors (TLR) on the surface of cells and interior of endosomes, and cytosolic NOD-like receptors (NLR). PRRs detect the presence of microbes through specific microbe-associated molecular patterns (MAMPs) such as peptidoglycan and lipopolysaccharide which are major constituents of bacterial cell walls [23]. The recognition of MAMPs by PRRs triggers the activation of several signalling cascades, among them NF-kB an important regulator of inflammatory cytokine production, as well as antimicrobial mechanisms to clear the infection. Mounting evidence supports a role for autophagy as an antimicrobial mechanism downstream of TLR and NLR signalling. For example, stimulation of NOD2 by muramyldipeptide (MDP), a constituent of peptidoglycan, induces autophagy in dendritic cells in a receptor-interacting serine-threonine kinase 2 (RIPK-2) dependent manner [24] and discussed in detail in a later section of this review, while stimulation of TLR2, 4 and 7 with their specific ligands has also been shown to stimulate autophagy [25,26]. In addition to its essential role in innate immune defence against infection, autophagy plays a role in the adaptive immune response. Autophagosomes can deliver pathogen protein fragments (peptides) to MHC class II molecules. Once the MHC class II molecule is bound to the pathogen fragment it is transported to the surface of the cell where it triggers an appropriate CD4^+^ T-cell response [27].

Recently, cargo receptor proteins or adaptors such as p62/SQSTM, NBR1 and NPD52 that contain both ubiquitin-binding and LC3-binding domains have been shown to detect intracellular bacteria [28]. Bacteria are heavily ubiquitinated in the host cell cytosol [29], therefore by simultaneously binding to ubiquitinated cargo and LC3-containing autophagosomes, cargo receptors can target invading microbes to lysosomes for degradation. For example, p62 and NPD52 have recently been shown to target the intracellular pathogen *Salmonella typhimurium* to the autophagic machinery [30]. Additionally, recent work has demonstrated that phosphorylation of the autophagy receptor optineurin by protein kinase TANK binding kinase 1 (TBK1) enhanced the LC3 binding affinity and autophagic clearance of ubiquitin-coated cytosolic *Salmonella enterica* [31]. While it is clear that both PRRs and cargo receptors contribute to the detection and elimination of cytosolic bacteria via autophagy, it remains to be determined how these factors functionally interconnect and whether they constitute a general anti-microbial defence mechanism or a more specific defence against pathogens.

## 4. Genetic Variants in Autophagy Pathway Genes and Crohn’s Disease Susceptibility

Before discussing the autophagy-related proteins implicated in CD aetiopathogenesis it is first important to recognise the genetic research that ignited the current interest in autophagy within the study of IBD. The first study to link the autophagy pathway with CD pathogenesis was a GWAS by Hampe *et al.* carried out in 2007 [32]. Using 735 CD patients and 368 controls, single nucleotide polymorphisms (SNPs) with a *p* value < 0.01 were followed up in three independent cohorts. This revealed a disease association with SNP rs2241880 in the autophagy-related 16-like 1 (ATG16L1) gene on chromosome 2q37.1. Resequencing of exons, splice sites and promotor regions concluded that the entire association signal was likely due to this threonine to alanine substitution (Thr300Ala or T300A), however very recent data has suggested additional variants, independent of rs2241880, could implicate any of the coiled-coil domain, the WD (tryptophan-aspartic acid, Trp-Asp or WD40 repeat) domain and/or the 3' untranslated region, in CD susceptibility [33]. Almost immediately following this initial finding, confirmation of this association was published by the same group in an independent cohort [34] and within 12 months multiple research groups had replicated the discovery in a variety of cohorts both in Europe and further afield [35,36,37,38,39]. Subsequently further individual genetic association studies have confirmed this finding in Caucasian populations [40], in addition to validation by several meta-analyses [41,42]. However replication in Asian populations has not been forthcoming despite various attempts [42,43,44,45]. Interestingly several paediatric studies have sought to confirm the association in childhood-onset CD however results have been conflicting, with two smaller studies confirming the association [46,47] while two larger studies proved negative [48,49], possibly reflecting the strong association with ileal Crohn’s disease (a phenotype not as common in childhood-onset disease [2]). 

Another gene on chromosome 5q33.1, immunity-related GTPase family, M (IRGM), was linked with CD susceptibility through a genome-wide association scan by the Wellcome Trust Case Control Consortium (WTCCC), again in 2007 [37]. In a case-control study involving 1748 CD patients and 2938 controls, SNPs achieving a *p* value < 10^−5^ (below the stringent threshold of their original study that incorporated several major diseases [50]) were followed up. Using a new panel of over a thousand Caucasian CD patients, allele frequency comparisons were made between CD cases and nearly six thousand non-autoimmune WTCCC cases (which included patients with bipolar disorder, coronary artery disease and hypertension) as well as independent population controls from the 1958 British Birth Cohort. The strongest replication adjacent to a known gene was for SNPs rs13361189 and rs4958847 immediately flanking the IRGM gene on chromosome 5. Resequencing of IRGM in 48 CD patients revealed three novel SNPs which were subsequently genotyped in an independent case-control cohort. This demonstrated that only the silent 313T > C variant was associated with CD. This SNP was in strong linkage disequilibrium with the original rs13361189 suggesting that the causal variant may lie in a regulatory region adjacent to IRGM thus affecting transcription. Since this initial finding, the association of SNPs in IRGM have been replicated in other adult CD populations [41,51,52], but again not in early-onset cohorts [53,54].

A subsequent meta-analysis of three GWAS with 3230 adult CD cases and 4829 controls demonstrated that the A allele of a SNP on chromosome 12q12 (rs11564258) was over-represented in CD patients, tagging a 0.89 Mb region containing both Leucine-rich repeat serine/threonine protein kinase 2 (LRRK2) and mucin 19, oligomeric (MUC19) [41]; this was later replicated in a paediatric GWAS [54]. Although the susceptibility gene at this locus has been shown to be most likely LRRK2 (rather than MUC19) [55], replication in subsequent individual studies has been inconsistent [56,57]. A further SNP (rs2412973) at locus 22q12 has shown association with childhood-onset CD patients [54]. With regards to colonic expression, the only gene in this region which was shown to differ between the two major IBD types (CD and ulcerative colitis) and healthy controls was myotubularin-related protein 3 (MTMR3). This gene showed significantly reduced expression in colonic biopsies in UC patients compared to controls and was also significantly associated with CD in the meta-analysis of discovery and replication cohorts. To date there have been no attempts to replicate this finding in either adult or paediatric populations.

Along with the original discovery of ATG16L1 variants, SNPs in the region of protein tyrosine phosphatase, non-receptor type 2 (PTPN2) were also described [37]. This was again replicated in several populations, including paediatric cohorts [58,59,60]. Genetic association studies have also identified SNPs in unc-51-like kinase 1 (ULK1) [61,62], with conflicting results with regard to the association of variations in neutrophil cytosolic factor 4 (NCF4) [35,63,64].

## 5. Autophagy Proteins Implicated in Crohn’s Disease Pathogenesis

### 5.1. ATG16L1 and NOD2

Following the initial identification and confirmation of ATG16L1 as a CD susceptibility gene in 2007 [32,35,36], and its relationship with an ileal disease phenotype [34], further studies began to unravel the potential biological relevance to this protein in CD pathogenesis. ATG16L1 is a member of a large group of ATG and ATG-related proteins which are intimately involved in autophagosome biogenesis [65]. Along with ATG12 and ATG5, ATG16L1 forms a 800 kDa complex in a 2:2:2 stoichiometry; although this ATG12 system has no deconjugating enzyme, the complex is formed constitutively irrespective of nutrient conditions [65,66]. During autophagosome formation the complex localises to the outer surface of the isolation membrane and dissociates following completion of the autophagosome [67]. 

One of the first studies that began to outline the precise functional role of ATG16L1 was by Fujita *et al*. [66]. Using a variety of mammalian cell culture techniques they demonstrated that the ATG12-ATG8-ATG16L1 complex was involved in LC3-lipidation, and interestingly that overexpression of ATG16L1 (specifically the coiled-coil region) disrupted the subunit stoichiometry leading to reduced autophagy. Also, the importance of correct localisation of ATG16L1 to sites of LC3-lipidation (such as the plasma membrane) was also shown to be vital for appropriate autophagosome formation. The same group later described the interaction of the Golgi-resident GTPase Rab33B with ATG16L1, however the precise pathways involved during autophagy are still to be determined [68]. More relevant to CD pathogenesis, Saitoh *et al*. demonstrated that ATG16L1-deficient macrophages produced high amounts of the inflammatory cytokines interleukin 1-beta (IL-1β) and interleukin-18 (IL-18) following stimulation with the Toll-like receptor 4 (TLR4) ligand lipopolysaccharide (LPS) [69]. They also showed that this increased IL-1β production was due to Toll/IL-1 receptor domain-containing adaptor inducing IFN-β (TRIF)-dependent activation of caspase-1 in ATG16L1-deficient cells. Additionally they utilised mice with ATG16L1-deficient haematopoetic cells to highlight their increased susceptibility to dextran sulphate sodium-induced colitis, which was in turn alleviated by treatment with IL-1β and IL-18 antibodies. 

Cadwell *et al*. extended this work by initially generating mice and murine embryonic fibroblasts that were hypomorphic for ATG16L1 (using gene trap-mediated disruptions of ATG16L1) [70]. Using this method allowed the detailed investigation of the consequences of low-expressing ATG16L1, avoiding the lethal effect of gene deletion [71,72]. They demonstrated that rapamycin-induced degradation of the adaptor protein p62 and LC3-II was diminished in ATG16L1 hypomorphic fibroblasts, which was restored by expressing ATG16L1. Additionally they showed that hypomorphic mice expressed approximately 25% of the expected levels of ATG16L1 in their ileum which equated to an increase in LC3-I:LC3-II ratio and p62. However, more importantly, these mice also showed striking abnormalities in Paneth cell morphology (Paneth cells are felt to be a key site in CD pathogenesis [73]) including lack of mucal lysozyme staining, reduced and disorganised granules, degenerating mitochondria and absence of apical microvilli. Following this, these changes in Paneth cell biology were confirmed in ileocolic resection specimens from CD patients carrying the ATG16L1 risk allele. Overall these data provided the first indication that ATG16L1 had a specific role in humans and mice in regulating the specialised properties of Paneth cells.

In 2010 there were several publications which intriguingly brought together two of the major pathways implicated in CD pathogenesis, namely the innate response (through NOD2) and autophagy. The innate immune system is the body’s first-line, non-specific response to foreign antigen. Both transmembrane and intracellular pattern recognition receptors (PRRs) recognise conserved, microbe-specific molecules named pathogen-associated molecular patterns (PAMPs; also known as microbe-associated molecular patterns (MAMPs)) such as LPS and peptidoglycan, leading to an appropriate immune response [23]. The NLR family are a major group of PRRs with a characteristic domain architecture comprising a central nucleotide binding and oligomerisation domain (NOD), an N-terminal effector binding (CARD) domain and C-terminal leucine-rich repeats (LRR) through which they detect MAMPs [74]. A key breakthrough in both CD and complex disease pathogenesis came in 2001 when fine mapping of the IBD1 locus on chromosome 16 identified the LRR variants of the NOD2 gene as conferring susceptibility to CD [75]. Since this discovery these NOD2 mutations have been widely replicated in both adult- and early-onset disease [41,54], with functional studies beginning to unravel the role of NOD2 in CD [76,77]. Although initially identified as a cytosolic protein, recent studies have shown that NOD2 also resides at the plasma membrane [78]. This membrane localisation suggests that NOD2 may function at the sites of bacterial entry to directly engage with pathogens and signal an appropriate inflammatory and antimicrobial response [78]. Furthermore, it is proposed that NOD2 can recognise danger associated molecular patterns (DAMPs) which are exposed when there is disruption of host cell membranes [79]. Some pathogens, such as *Salmonella typhimurium* and enteropathogenic *E.coli* (EPEC) use type-3-secretion systems (T3SS) to inject effector proteins into the cytosol of host cells to mediate adhesion and invasion, and this may act as a DAMP recognised by NOD2.

**Figure 3:**
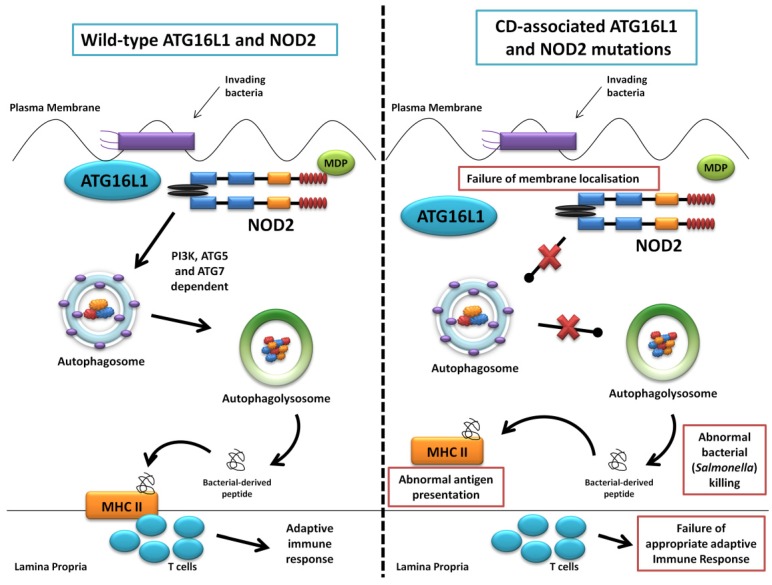
Summary diagram showing the role of ATG16L1 and NOD2 in the autophagy pathway and the defects observed when Crohn’s disease (CD) associated mutations are present. The left panel shows the normal autophagy response with NOD2 recruiting ATG16L1 to the plasma membrane, normal autophagosome and autophagolysosome formation, followed by MHC II antigen presentation and an appropriate adaptive immune response. The right panel demonstrates abnormal membrane localisation of ATG16L1 with the LRR and T300A mutations resulting in abnormal bacterial killing and defective antigen presentation.

The first of the seminal papers involving ATG16L1 and NOD2 demonstrated that NOD2 stimulation by its ligand muramyl dipeptide (MDP) induced autophagy in human, monocyte-derived dendritic cells (DCs) and also influenced bacterial handling and antigen presentation [24]. This was shown to be independent of TLR4 signalling and required the NOD2 signalling mediator RIPK-2 in addition to PI3K and the autophagy proteins ATG5, ATG7 and ATG16L1. Additionally, they demonstrated that DCs isolated from individuals with any of the three known CD-associated NOD2 or T300A ATG16L1 variants exhibited defective autophagy. Also cells carrying a NOD2 variant failed to localise *Salmonella enterica* and CD-associated adherent-invasive *E. coli* species to autophagosomes, with this effect reversed by artificially inducing autophagy using rapamycin. At the same time a study led by Dana Philpott in Toronto similarly used bone marrow-derived macrophages and macrophages from NOD2-deficient mice to demonstrate NOD2-dependent autophagy induction with MDP [80]. Interestingly this group highlighted RIPK-2 independent autophagy induction, with colocalisation of NOD2 and ATG16L1 at the plasma membrane, with only cells homozygous for the T300A mutation effecting autophagy. NOD2 and ATG16L1 were also found to surround invading pathogens at the entry foci with mutant NOD2 proteins failing to do so. Homer *et al*. further showed that MDP-activated autophagy and NF-kB signalling, in addition to increased *Salmonella* killing, was dependent on NOD2 and ATG16L1 expression, specifically in intestinal epithelial cells; this response was again shown to be altered by known CD-associated NOD2 mutations [81]. The same group has most recently show the dual role of RIPK-2 in NOD2-induced autophagy with a positive signal through activation of p38 MAPK and reduced autophagy mediated by the phosphatase PP2A [82]. Kuballa *et al*. also showed abnormal capture of internalised *Salmonella* within autophagosomes in epithelial cells carrying the T300A mutation [83]. A summary of the role of ATG16L1 and NOD2 in autophagy and the defects demonstrated when CD-associated mutations are present are shown in Figure 3.

Further work has now shown that ATG16L1 is intimately involved in autophagosome formation with Ravikumar *et al.* showing that ATG16L1 interacts with the heavy chain of clathrin (a protein that plays a major role in the development of coated vesicles [84]) during the formation of ATG16L1-positive pre-autophagosomes [85]. This was shown to occur at the plasma membrane, although no obvious disruption of the clathrin-ATG16L1 interaction was observed with the T300A mutation. The inflammatory cytokine profile of cells possessing the T300A mutation has also been investigated with MDP-stimulation of peripheral blood cells harbouring T300A showing increased IL-1β, IL-6 and IL-10 [86,87], in addition to a reduction in IFN-g production [88]. Lee *et al*. went on to show that increased IL-1β production was a result of increased p62 levels in ATG16L1-deficient cells [89]. The T300A mutation has also recently been shown to produce hyperstable interactions between DCs and T cells at the immune synapse, with increased T cell activation, specifically the T-helper 17 cell response [90]. Work highlighting the role of ATG16L1 during *Helicobacter pylori* infection as well as murine norovirus has also been presented [91,92].

### 5.2. IRGM

The immunity-related GTPase (IRG) family of proteins, first described in the 1990s, can be separated into IRGA, IRGB, IRGC, IRGD and IRGM subfamilies based on homology across their GTP-binding domain, however only two *IRG* sequences, both transcribed, are present in humans, namely *IRGC* and IRGM [93]. IRGC is not induced by interferons (unlike IRGM) and seems to be very tissue-specific, having been shown to be mainly expressed in the testes [93]. To date, five different 3'-splicing isoforms have been identified for human IRGM with the protein products of these isoforms predicted to have molecular weights of between 19–24 kDa [94].

Interferon-gamma (IFN-g) has long been recognised as important cytokine during the cellular response to bacterial invasion [95]. One of the first indications that the 47-kDa GTPase family, including IRGM, was involved in this response was the observation that genes encoding these proteins were upregulated during IFN-g stimulation [96,97]. Culture of murine macrophage and fibroblast lines in medium containing high concentrations of IFN-g showed a significant number of cDNA fragments belonging to both the 65-kDa and 47-kDa GTPases, including Irgm1 (also known as LRG-47, the murine homologue of IRGM). Following this, work by MacMicking *et al*. demonstrated that Irgm1 null mice failed to control *Mycobacterium tuberculosis* replication, with defective bacterial killing of *mycobacterium*-containing phagosomes in null macrophages [98]. MacMicking *et al*. also observed impaired maturation of *Mycobacterium tuberculosis*-containing phagosomes suggesting a critical role of Irgm1 in vacuolar trafficking; this work was validated in a similar study by Feng *et al*. [99]. A further study investigated this role showing that there was an increase in autophagic vacuoles and the maturation of mycobacterial phagosomes in murine macrophages transfected with Irgm1, with Irgm1 partially co-localising with small LC3-positive organelles [100]. Singh *et al.* used a number of autophagic markers such as monodalsyncadaverine (MDC), Lyso-Tracker (LT) red and Mito-Tracker (MT) red to demonstrate early autophagic vacuoles, vacuole acidity and the presence of mitochondrial material in vacuoles respectively, induced by over-expressing Irgm1 in murine macrophages [101]. However, more interestingly, they also demonstrated that human IRGM participates in autophagy induced conventionally (by the use of rapamycin or serum starvation) and by IFN-g in human macrophages, and confirmed an increase in mycobacterial survival using siRNA to IRGM. More recent work has also shown that IRGM participates in virus-induced autophagy, with siRNA knockdown of IRGM decreasing the number of autophagosomes induced by measles virus, hepatitis C virus and HIV proteins [102]. Although many other studies have confirmed the importance of IRGM in cellular resistance to a number of pathogens [103,104] some conflicting evidence still exists [105]. 

### 5.3. LRRK2

Leucine-rich repeat serine/threonine protein kinase 2 (LRRK2; also known as PARK8) is a multi-domain protein of 2527 amino acids [106]. This large ubiquitous protein with multiple functional domains, namely kinase, ROC GTPase, Cor, leucine-rich repeat, ankyrin and WD40 [107] has been linked to CD through the association of a SNP on chromosome 12q12 [108]. LRRK2 is expressed in a large number of murine tissues, including lung, heart, kidney and small intestine [109]. In addition, recent work has demonstrated that full length LRRK2 is a common constituent of human peripheral blood mononuclear cells such as CD14^+^ monocytes, CD19^+^ B cells and CD8^+^ and CD4^+^ T cells [110]. 

To date, little work has focussed on the potential functional role of LRRK2 in CD pathogenesis. However, due to mutations in LRRK2 having been consistently associated with Parkinson’s disease (PD) (a neurodegenerative disease characterised by progressive disturbances in motor, autonomic and psychiatric functions [111]) an increasing number of studies are now beginning to unravel the complex functions of this protein. The best characterised mutation in LRRK2 (G2019S—a glycine to serine substitution at amino acid 2019) was initially demonstrated in several familial cases of PD [112] and subsequently common variations in LRRK2 have been shown to contribute to the risk of sporadic PD [113,114]. This, coupled with the previously established importance of autophagy in a number of neurodegenerative diseases [115], has focussed current research on the role of LRRK2 during the autophagic process. 

LRRK2 has been shown to localise to vesicular granular structures of the late endosomal-lysosomal pathways [116,117,118]. Several membrane microdomains such as the neck of caveolae, microvilli/filopodia and intraluminal vesicles of multivesicular bodies (MVBs) were shown to contain LRRK2, as well as cytoplasmic puncta corresponding to MVBs and autophagic vacuoles [116]. A recent study demonstrated that overexpression of wild-type LRRK2 increased autophagy through activation of a calcium-dependent protein kinase kinase-β (CaMKK-β)/adenosine monophosphate (AMP)-activated protein kinase (AMPK) pathway [119]. This effect was through nicotinic-acid adenine dinucleotide phosphate (NAADP) receptors causing calcium efflux and partial alkalinisation of lysosomal store pH. Increased LC3 punctae were also visualised with overexpression of full-length LRRK2, but not the mutant protein, suggesting involvement of the kinase domain. Further work has provided evidence that the presence of the G2019S-mutant protein in neuronal cells leads to the accumulation of autophagic structures, with an impaired autophagic balance also demonstrated in non-neuronal and yeast cells [119]; induced pluripotent stem cells from patients with the G2019S mutation also show an accumulation of autophagic vacuoles [120]. Similarly, aged LRRK2-null mice show increased LC3-II and p62 in the kidney with a subsequent inflammatory response [121].

More pertinent to CD pathogenesis, work by Liu *et al*. showed that LRRK2-deficient mice had significantly poorer clinical outcomes in an experimental (DSS-induced) colitis model compared to their wild-type counterparts, manifested as increased weight loss and diarrhoea [122]. Further work by Gardet *et al.* in human subjects used peripheral blood mononuclear cells and intestinal biopsies from IBD patients to further delineate the role of LRRK2 [123]. Stimulation of macrophage-differentiated THP-1 cells with IFN-g showed an increase in both LRRK2 mRNA and protein, with PBMCs from healthy controls showing a similar response in CD3^+^ T cells, CD19^+^ B cells and CD11b^+^ monocytes. LRRK2 mRNA expression was also shown to be increased 6-fold in inflamed CD biopsies compared to paired non-inflamed biopsies, with histological specimens showing LRRK2-positivity in CD206 macrophages, CD103^+^ dendritic cells and CD20- B cells. Gardet *et al*. also showed using luciferase-based reporters that LRRK2 can activate NF-kB pathways dependent on the IKK complex. Particularly relevant to CD they also demonstrated that LRRK2 co-localised with *S. typhimurium* in the cytosol of RAW macrophages, with LRRK2 siRNA knockdown reducing reactive oxygen species after IFN-g stimulation and increased *S. typhimurium* survival in a gentamicin-protection assay. Extending this work, Hakimi *et al*. showed that LRRK2 is present in a subset of circulating leucocytes with the expression of ^R1441C^mutant LRRK2 in a primary, non-neural cell model revealing an autophagy defect manifested as a reduction of LC3-II levels [110]. Stimulation of murine bone-marrow derived macrophages by a variety of pattern-recognition receptor ligands (which are likely to be pivotal in CD pathogenesis [124]) produced up-regulation of LRRK2 mRNA with respect to LPS, R837 and CpG and down-regulation when stimulated with Pam3CSK4. 

### 5.4. MTMR3

MTMR3 encodes a protein of the tyrosine phosphatase superfamily [125]. The MTMR proteins are involved in the regulation of phosphatidylinositol (PtdIns) and its derivatives, accounting for around 10% of the total lipid in eukaryotic membranes [126]. This family of proteins (a subgroup of the PtdIns(3)*P* phosphatases) has 15 members, only nine of which (including MTMR3) possess the active phosphatase domain that specifically dephosphorylates PtdIns(3)*P* [127]. PtdIns(3)*P* is highly enriched as a component of the elongating isolation membrane and autophagosome membranes in yeast [128], with MTMR3 ubiquitously expressed and shown to hydrolyse PtdIns(3)*P* to its derivative PtdIns [129]. Roles in autophagosome size and constitutive autophagy initiation in epithelial cells [130], coupled with hVps34 (a class III PI 3-kinase) being intimately involved in autophagy and the suppression of autophagy by the PI 3-kinase inhibitor Wortmannin, makes this group of proteins of particular interest [131,132]. PtdIns(3)*P* has specifically been shown to bind to the autophagy protein ATG18 [133,134,135] and this ATG18-PtdIns(3)*P* interaction has now been recognised as an essential component for the efficient progression of both selective and non-selective autophagy [136]. It is likely that the ATG18-PtdIns(3)*P* complex exerts this role through the interaction of the ATG18-ATG2 complex with ATG9 which is the only known integral membrane ATG protein [137]. Further work has now extended the role of MTMR3 specifically in autophagy. Work in alveolar basal epithelium (A549 cells) by Taguchi-Atarashi *et al*. showed that overexpression of mutant MTMR3 (MTMR3^C413S^) produced an increase in GFP-LC3 and GFP-ATG5 which was not seen with wild-type overexpression [131]. Also, this increase in autophagy was dependent on PtdIns(3)*P*, with siRNA knockdown of MTMR3 producing a similar response, suggesting MTMR3 as a negative regulator of autophagy. The 22q12 locus harbouring MTMR3 has now also been associated with lung cancer [138], with specific MTMR3 mutations discovered in gastric and colorectal cancer [139], all of which may be important in the context of the emerging role of autophagy in cancer biology [140].

### 5.5. PTPN2

The PTPN2 gene is located on chromosome 18p11 and codes T cell protein tyrosine phosphatase (TC-PTP, also known simply as PTPN2), one of over 100 PTP proteins capable of removing phosphate moieties from tyrosine residues of protein substrates [141]. First cloned in 1989 [142], this ubiquitous protein exists as two splice variants, one localised to the nucleus and the other to the endoplasmic reticulum [141]. Much of the functional work surrounding this protein has been through investigation of PTPN2 knockout mice that demonstrate a progressive systemic inflammation and abnormal cytokine environment [143]. TC-PTP has also been shown to be involved in haematopoiesis and autoimmune disease [141]. 

Much of the work specifically relating to the role of PTPN2 and CD has been performed by Scharl *et al*. The first of these papers studied the role of PTPN2 in intestinal barrier function. They demonstrated that PTPN2 was over-expressed in biopsies from CD patients, and that stimulation of an intestinal epithelial cell (IEC) line with IFN-g increased PTPN2 levels [144]. Also, PTPN2 knockdown increased STAT1 and STAT3 phosphorylation after IFN-g stimulation and increased epithelial permeability as determined by FITC-Dextran flux across IEC monolayers. Similar increases in IL-6 and macrophage chemoattractant protein 1 (MCP-1) were seen with PTPN2 knockdown in a monocyte cell line in a subsequent study [145]. Other work showed comparable results when IECs were stimulated with TNF-α [146]. Very recent research has now shown a role for PTPN2 in autophagy with siRNA knockdown in IECs reducing autophagosome formation as well as autophagy induction as determined by persisting *Listeria* infection; this was replicated in colonic lamina propria fibroblasts from patients with CD carrying the PTPN2 variant [147]. Additionally a novel variant in PTPN2 (rs1893217) was also shown to impair autophagosome formation, as well as elevating IFN-g levels in response to MDP [148].

### 5.6. NCF4 and ULK1

One of the first GWAS to identify the association of autophagy genes with CD susceptibility postulated NCF4 as a candidate gene [35]. Although this finding was replicated soon afterwards [63], several studies have also proved negative [64,149]. NCF4 (also known as p40phox) is a member of the cytoplasmic SH3-domain proteins involved in the activation of gp91phox during phagocytosis [150]. Along with other phox proteins (e.g., p67phox) NCF4 is involved in the generation of reactive oxygen species (ROS) by the reduced nicotinamide adenine dinucleotide phosphate (NADPH) oxidase complex, which is critical in the antimicrobial functions of phagocytic cells [151]. In addition to NCF4-deficient mice showing defective killing of *S. aureus* [151], ROS have also been shown to be important during autophagy induction [152]. A limited number of studies have demonstrated an association with an ileal [63,153] or perianal [154] phenotype with one functional study showing a reduction in reduced ROS in GM-CSF-primed granulocytes from CD patients harbouring the NCF4 mutation [155]. 

To date only two studies have evaluated the role of the well-characterised autophagy protein ULK1 in CD pathogenesis. The first of these demonstrated that the SNP rs12303764 near ULK1 was significantly associated with CD in a case-control study, replicated in a transmission disequilibrium test in 335 case-parent trios [61]. This association with ULK1 variants was then recently replicated in an independent case-control study in New Zealand [62]. Although the association with rs12303764 was not present in their population, several other SNPs in the region of ULK1 showed positive association, however the replication for these SNPs in larger GWAS meta-analysis data was variable. The ULK1 gene, consisting of 28 exons, with a transcript length of 5211 basepairs and a translation length of 1050 residues, is localised on chromosome 12q24. The two known mammalian orthologs of yeast Atg1, ULK1 and ULK2, form a stable complex with ATG13, FIP200, ATG101 and mTORC1. Although this complex is intimately involved in autophagy induction, in view of its currently weak association with CD a detailed discussion of the role of ULK1 in autophagy is outwith the scope of this article; however several excellent reviews of its biological function are available [156,157]. 

**Figure 4:**
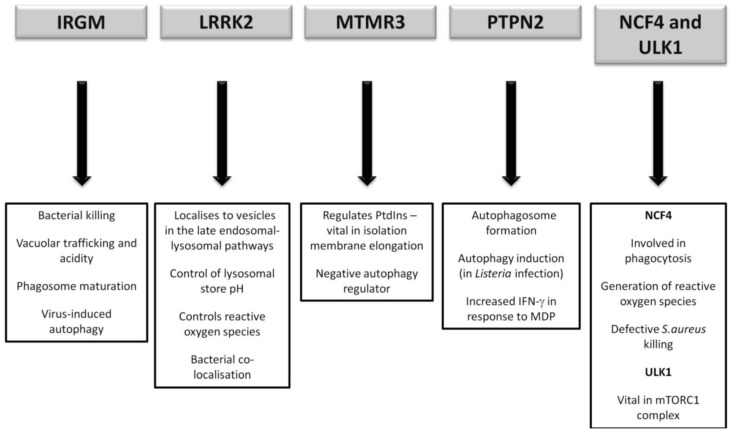
Postulated autophagy-related pathways influenced by CD susceptibility proteins currently under more detailed investigation.

It has yet to be seen what direct role CD-association variants in NCF4 and ULK1 will play, if any, in CD pathogenesis. However future research will undoubtedly endeavour to illicit any further association given the vital role of ULK1 in the mTORC1 complex and the intriguing importance of ROS in bacterial handling. A summary of the postulated autophagy-related pathways influenced by the lesser-studied CD susceptibility proteins are outlined in Figure 4.

## 6. Adherent Invasive *E. coli*

*E. coli* is normally a harmless commensal micro-organism that can become a highly-adapted pathogen through the acquisition of specific virulence factors. To date eight pathovars have been extensively studied and implicated in a wide range of diseases, of which enteropathogenic *E. coli* (EPEC), enterohaemorrhagic *E. coli* (EHEC), enterotoxinogenic *E. coli* (ETEC) and diffusely adherent *E. coli* (DAEC) cause gastroenteritis in humans [158]. In addition there are several recently described *E. coli* pathovars, of which AIEC are the most relevant to CD. The association of AIEC with CD was first reported by Darfeuille-Michaud and colleagues in the late nineties [159,160], and since then AIEC have been isolated from independent studies involving adult and paediatric CD patients [7,8,161]. The high prevalence of AIEC strains observed in the ileal mucosa of CD patients has been attributed to their ability to adhere to the glycosylated carcinoembryonic antigen-related cell adhesion molecule 6 (CEACAM6), which is overexpressed on the surface of epithelial cells in patients with CD [162]. In addition the ER-stress response chaperone Gp96 is highly expressed in epithelial cells of patients with CD and has been demonstrated to act as a host cell receptor for AIEC [163]. Recently, complete genome sequencing of the reference strain of CD-associated AIEC LF82 has shown that this strain contains several virulence factors including the *lpf* operon, which encodes long polar fimbriae (LPF) [164]. AIEC have also been shown to interact with Peyer’s patches, specialised cells of the intestinal epithelium via LPF, and the prevalence of AIEC strains containing the *lpf* operon was shown to be markedly higher in CD-patients compared to controls [165]. 

Following adhesion AIEC can invade the intestinal epithelium, breaching the epithelial barrier and are subsequently found in the lamina propria of patients with ileal CD. A recent study investigated the role of autophagy in the replication and survival of *E. coli* in epithelial cells [166]. Specifically, the authors investigated whether cells deficient in the CD-associated autophagy proteins IRGM and ATG16L1 affected intracellular replication and survival of AIEC strains. This study demonstrated that the AIEC strain LF82 have enhanced replication and survival in both IRGM and ATG16L1 deficient cells. Remarkably, autophagy deficiency did not interfere with the replication and survival ability of other *E. coli* strains tested, including non-pathogenic, environmental, commensal, or pathogenic strains involved in gastroenteritis, suggesting a specific role for autophagy in restraining AIEC. AIEC can translocate the epithelium into the lamina propria where they can replicate and survive within macrophages. In a subsequent study the same group investigated whether defects in autophagy affected the replication and survival of AIEC in macrophages [167]. AIEC were shown to recruit the autophagy machinery to sites of entry into host cells and autophagy limited intramacrophagic replication. Impaired IRGM or ATG16L1 expression resulted in increased AIEC in macrophages and significantly this was accompanied by increased secretion of the pro-inflammatory cytokines IL-6 and TNF-a. Further studies demonstrate that a family of micro-RNA (miRNAs) miR-196 that are expressed in the intestinal epithelium of individuals with CD down regulate the expression of IRGM in those with the protective variant (c.313C) [168]. The resulting impairment of the autophagy response results in increased intracellular replication and survival of AIEC. Taken altogether these studies suggest that stimulation of autophagy, perhaps using small molecule inhibitors of mTORC1, would be a therapeutic strategy to restrain AIEC replication and dampen the exacerbated inflammatory response that leads to chronic inflammation and granuloma formation observed in patients with CD. 

## 7. Autophagy Pathway-Phenotype Correlations in Crohn’s Disease

A number of studies have now endeavoured to correlate CD phenotypes with autophagy gene risk variants in an attempt to translate knowledge of the autophagy pathway to clinical application. The most replicated finding in this respect is the positive association of ATG16L1 and IRGM variants with ileal disease phenotype. Along with Prescott *et al*. who initially reported this finding for ATG16L1 in a cohort from the United Kingdom [34], groups in Australia [169] and Portugal [57] have recognised this relationship, with the Portuguese group also showing this association for IRGM. Similarly carriage of the ATG16L1 risk variant has been shown to correlate with a reduction in extra-intestinal manifestations; however the small patient numbers in this report may have been a confounding factor [170]. Another reported positive association was with IRGM variants and the need for ileocolectomy (a surgical procedure to remove the affected distal small bowel and proximal large bowel), however the number of patients in the study again make this difficult to interpret [171]. Several recent studies have attempted to define the association of autophagy gene risk variants with the presence of granulomas on intestinal biopsy samples, with conflicting results. (A granuloma is an inflammatory lesion containing epithelioid cells, macrophages, and lymphocytes, and is considered one of the pathological hallmarks of CD, however detection at endoscopy is variable [172]). Wolfkamp *et al.* in a cohort of approximately 200 patients demonstrated no association of granulomas with CD-risk variants in ATG16L1 or IRGM [173]. SNPs in NCF4 and *ATG4A* have also shown negative correlation with the presence of granulomas in an Israeli cohort [174]. A larger study from Belgium, which included 464 patients, did find both a positive and negative association of variations in four autophagy-related genes (ATG2A, ATG4A, ATG4D and FNBP1L); to date these genes have not been shown to confer a susceptibility risk to, or altered protein expression in, CD [175].

The role of the microbiome in the pathogenesis of CD has become more prominent in recent years [5], a small number of studies have aimed to determine the relationship between autophagy risk-variants and microbial components. Murdoch *et al*. looked at the presence of four antimicrobial antibodies stratified by CD-associated variants [176]. They showed that in a cohort over 600 CD patients, that increased seropositivity for anti-Saccharomyces cerevisiae antibody (ASCA) was associated with carriage of the ATG16L1 variant and that the IRGM CD-risk variant was associated with increased anti-flagellin seropositivity. Frank *et al*. looked at DNA extracted from intestinal specimens from 35 CD patients to determine the microbial composition and its relationship with genotype [177]. 16S rRNA sequence analysis revealed that patients with the NOD2 or ATG16L1 variants had significantly altered microbiota, although the authors did acknowledge that the study was underpowered to determine any genera-specific effects. 

## 8. Conclusions

It can be seen that following the emergence of autophagy as a pathogenic mechanism in CD that some progress has been made to unravel the functional aspects of these complex pathways. To date much of the research surrounding this topic has been focussed on genetic susceptibility and despite the field of autophagy progressing at a rapid pace, much is still unknown regarding the precise molecular abnormalities that may underlie the disease process. It is likely that further knowledge of the autophagy proteins involved specifically in CD and a deeper insight into the role of xenophagy (especially in the context of AIEC and the ileal disease phenotype) will be required before effective intervention can be implemented to alter the disease course. Functional work is now required to determine the potential therapeutic modulation of both the autophagy pathway itself, in addition to targeting specific bacterial subgroups such as AIEC, although these therapies will most likely only be beneficial in a small number of patients.
